# Gun-cotton

**Published:** 1846-12

**Authors:** 


*Gun-cotton.—Xyloidine.*—It is rather more than two months since we inserted a notice of a remarkable chemical discovery reported to have been made by Prof. Schönbein, of Basle. We allude to the preparation of cotton so as to give it fulminating properties, and to render it a safe, inexpensive, and simple substitute for gunpowder. We then announced it as probable that the Professor would give a full account of his alleged discovery at the meeting of the British Association of Southampton. To the surprise and disappointment of all scientific men, this meeting was converted into an advertising medium for the so-called gun-cotton; and the Professor declined to give the least intimation respecting the preparation of the substance, as it was his intention to take out a patent for it, and thus render it a commercial speculation. After the noble example of Sir H. Davy, who declined to patent his safety-lamp, we should have thought scientific men would have hesitated before resorting to the patent laws for a pecuniary remuneration; and we certainly think that the British Association committed a grave error in allowing the subject to be brought publicly forward, when there was no intention, on the part of the alleged inventor, to describe the process by which the gun-cotton was prepared.

Within the last week, public attention has been much directed to the subject. It is reported that the German Diet has conditionally awarded 100,000 florins as a reward to the inventor. The *Athenæum* informs its readers that a hundred weight of the gun-cotton is now on its way from Basle to Woolwich, having been ordered by our government with a view of testing its applicability to heavy ordnance.

In the meantime, although it does not appear that Professor Schönbein had divulged his secret, Dr. Otto, Professor of Chemistry in Brunswick, has addressed a letter to the *Hanoverian Gazette*—since published in the *Times*—in which he states that he was led, from the researches of Pelouze, to infer that the cotton was soaked in nitric acid of a certain strength, washed, and dried. Thus the secret of the gun-cotton became at once public. On the 4th of October, Dr. Otto performed certain experiments with his preparation, the results of which satisfied him that it must be identical with the gun-cotton of Schönbein. At a late meeting of the Academy of Sciences, in Paris, M. Arago gave an account of certain experiments performed with prepared cotton by a M. Morel, the results of which satisfactorily showed that it was capable of forming an admirable substitute for gunpowder; and, with all that enthusiasm which characterises our Gallic neighbours, M. Arago pictured an army entering on a campaign, with a few bales of cotton and a few gallons of nitric acid, making their own explosive cotton as they required it! M. Morel, it is stated, has secured a patent for France; and, so far as we can ascertain, he has acquired his knowledge of the subject independently of any communication from M. Schönbein. The latest intelligence is, that the last mentioned gentleman has procured a patent for England and her colonies.

Having thus given a slight history of what has transpired publicly on this subject, we now propose to consider how far M. Schönbein has a claim to be regarded as the inventor of gun-cotton, assuming that he employs nitric acid like Dr. Otto and M. Morel.

About six or seven years since, it became pretty generally known to the chemists of England, from the researches of M. Pelouze, that when woody fibre, whether as paper, sawdust, or linen, was saturated with strong nitric acid, washed, and dried, its properties were considerably altered. A principle called *xyloidine* was produced; and the woody fibre, although all the acid was washed out of it, burnt rapidly, and often with explosive violence. We saw this experiment made about six years since; but, from that time, the subject appears to have received from chemists no particular notice, until the alleged invention of Schönbein recalled the attention of Dr. Otto and others to the researches of Pelouze.

In various chemical works published in 1842–3, the action of nitric acid on woody fibre is especially mentioned. Thus, in Turner's Chemistry, (ed. 1843, p. 1222), it is stated, in reference to woody fibre, "In strong nitric acid saw dust dissolves; and, on the addition of water, a white insoluble powder is deposited, which contains nitric acid, and explodes when heated." In Graham's Chemistry (ed. 1842, p. 295), the facts are more explicitly stated, as the following extract will show:—"Nitric acid, in its highest state of concentration, exerts no violent action upon certain organic substances, such as lignin or woody fibre and starch, for a short time, but unites with them, and forms singular compounds. A proper acid for such experiments is procured with most certainty by distilling 100 parts of nitric acid with no more than 60 parts of the strongest oil of vitriol. [These are exactly the proportions recommended by Dr. Otto.] If paper is soaked for one minute in such an acid, and afterwards washed with water, it is found to shrivel up a little, and become nearly as tough as parchment, and, when dried, to be remarkably inflammable, catching fire at so low a temperature as 356°, and burning without any nitrous odour, (Pelouze)."

Professor Graham here, it will be seen, gives in 1842, an outline of Pelouze's discovery, and, by the substitution of cotton for paper, it becomes the so-called discovery of another in 1846!

We shall now give an extract from the Traité de Chimie of M. Dumas. At page 12, tome vi., published in 1843, this author says: "When cloth, (either linen or cotton,) or a sheet of paper is soaked for a few minutes in nitric acid of a specific gravity of 1.4, and afterwards washed in water, the xyloidine formed at the expense of a part of the vegetable tissues remains locked up in the fibre, rendering the paper and the cloth impermeable to water, and much more combustible. These properties suggested to M. Pelouze the idea of employing them in the manufacture of cartridges for artillery."

M. Schönbein may have known nothing of Pelouze's researches; nevertheless, it appears to us clear that Pelouze was the real and original inventor of the process for preparing gun-cotton. His countryman, M. Dumas, informs us that he even announced the plan of applying these substances to the very purpose for which M. Schönbein has taken out a patent.

With respect to the process for preparing this substance, we have found that the acid best adapted for the purpose is that recommended by Pelouze. It is obtained by distilling ten parts of dry nitre with six of sulphuric acid ; and it is strictly speaking the acidum nitrico-nitrosum. The cotton wadding should be thoroughly steeped in this acid for about three minutes, then plunged into water, and washed under a current, until litmus paper is no longer reddened by the washings. The cotton should be well squeezed in a cloth—picked out and gently dried before a fire. It requires some time to dry a mass of it thoroughly; since the porous material is very retentive of water. When at all humid it burns slowly and without explosion. When dry it burns suddenly with a bright yellow flame, a feeble detonation, and leaves no residue. Compared with its bulk its explosive properties do not appear remarkable: but when compared with its weight they are greater than those of gunpowder. The explosion is less rapid that that of the fulminating compounds of the metals ; but more rapid than that of gunpowder. When well prepared it explodes at a very moderate heat (about 420°,) gives off scarcely any visible smoke, and leaves no residue. This substance has a great advantage over gunpowder in the fact that when well prepared the whole of it is dissipated in gas—carbonic acid and nitrogen. With gunpowder there is always a residue of carbon and sulphuret of potassium,—the latter tending to corrode the metal. Experiment only can determine the relative gas-producing powers of the two substances. The gun-cotton may be inflamed over gunpowder without igniting it. There is nothing extraordinary in this : hydrogen may be inflamed in contact with gunpowder without kindling it, and a small quantity of alcohol may be burnt over it with a like negative result. Gunpowder requires a red heat for its ignition, and unless one particle of the mass reaches this temperature it does not explode. There can be no doubt that the gun-cotton explodes at a much lower temperature than gunpowder, and as percussion will produce the same effect as heat, it should be cautiously handled when thoroughly dry.

It is altogether a remarkable substance; and may, upon trial, entirely supersede gunpowder. Should this be the case, it may have the effect of rendering nitric acid cheaper and more abundant, since the employment of nitre for the manufacture of gunpowder would be no longer necessary.

We have prepared this substance with the strongest nitric acid 1.52,—with the acid at 1.4, distilled as above stated, and with a mixture of nitric and sulphuric acids; but, according to our experiments, the most certain and satisfactory process is that above described. The cotton is undoubtedly highly oxygenized, and it may be worth inquiring, whether in a highly dried condition it may not be liable, in masses, to spontaneous combustion. It might be hazardous to keep a large store of it completely dried. The presence of a very small quantity of moisture is sufficient to counteract its explosive properties ; and as cotton from its porousness is very hygrometric, it will be proper to consider how far this property may interfere with its employment as a substitute for gunpowder. If it be true as it is reported, that the government have ordered a hundred weight of this substance from Basle, in Switzerland, the cost of transport will far outweigh the cost of the materials. This quantity might have been made at Woolwich under the superintendence of M. Schönbein, and rendered fit for use at a small expense in the course of a few hours.

In conclusion we shall observe, that although we think the merit of the discovery is due to M. Pelouze, yet M. Schönbein deserves credit for having at least called public attention to the subject. He has, however, been the involuntary means of making the practical value of M. Pelouze's researches well known. His secret has transpired in spite of his attempt to conceal it. *Suum cuique.—London Medical Gazette.*

